# Case of Acute Rheumatism Translated to the Heart

**Published:** 1823-03

**Authors:** Andrew Armstrong

**Affiliations:** Assistant Surgeon, Grenadier Regiment of Foot Guards.


Art. VI.—*Case of Acute Rheumatism translated to the Heart.* By Andrew Armstrong, Esq. Assistant Surgeon, Grenadier Regiment of Foot Guards.

On the 7th of November, Thomas Pinder, private soldier in the grenadier regiment of Guards, a young man, of fair complexion and slender form, was admitted into the regimental hospital with acute rheumatism, characterized by very severe pain in the wrists extending along the fore-arms, violent pyrexia, a full and hard pulse, great thirst, furred tongue, and a costive state of the bowels. Twenty-four ounces of blood were immediately taken from his arm in a full stream; he was ordered a brisk cathartic, and put on fever diet.

On the morning of the 8th, he was found to have passed a sleepless night, the pain in the joints being very severe: indeed, none of the symptoms were milder. His bowels had been freely opened; and the blood exhibited the sizy appearance denoting acute local inflammation in a high degree. He was again bled to the same extent as the preceding day, and ordered small doses of tartarized antimony, to the extent of one-fourth of a grain every three hours.

On the 9th, the symptoms appeared a little milder, though still very urgent. The antimonial medicine was continued, and he took ten grains of the compound powder of ipecacuanha at night. The appearance of the blood was as inflammatory as before.

On the 10th, he complained for the first time of cough, but it gave him scarcely any inconvenience. His bowels had not been moved the preceding day; and the pains in the joints and fever were undiminished. A brisk cathartic, of calomel and compound extract of colocynth, was again prescribed; which was to be followed up with half an ounce of Epsom salts every two hours, until the bowels had been well opened.

On the 11th, his symptoms continued very severe, although the opening medicines had fully answered the purpose. The cough was more frequent, and he complained of its being attended with pain in the left side. He was again bled, and continued the use of the tartarized antimony, with the intention of producing a diaphoretic effect.

12th.—His pulse had acquired a degree of strength and fulness exceeding any thing of the kind that Mr. Watson, the surgeon on duty, or I myself, had ever met with before. It was now thought proper, as affording him the only chance, to bleed him either *ad deliquum*, or, should this not happen, to a very great extent. Forty ounces of blood were taken very rapidly from the arm, from a large orifice. Syncope, however, was not produced; nor was his pulse considerably affected by it. He took the following mixture:

℞. Liquor. Antimon. Tartariz. ʒij.
——— Ammon. Acet. ℥ij.
Aquæ, ℥vj. M. ℥jss. 3tia quaque hora sumend.

On the 13th, he had passed another sleepless night, from the violence of the pain in his arms. His cough, and the pain in the left side, which he referred exactly to the situation of the heart, had also increased. His bowels were again disposed to be confined, and the arterial action was but little diminished. A purging enema was thrown up, and he continued the use of the mixture, to which forty drops of the tincture of digitalis was added. He previously took another cathartic.

At the evening visit, the disease was evidently gaining ground. He complained more of the pain of the left side, and his pulse was as hard and full as ever, vibrating most powerfully under the finger. Thirty-six leeches were applied to the left side, with instructions to encourage the bleeding; and the dose of the digitalis was increased.

On the 14th, he was considered rather better, and hopes were entertained that there was still a chance of his recovery, as the pains in the arms were milder, and he complained less of the pain in his side. These hopes were, however, of short duration: he was seized with fainting-fits whenever his head was raised from the pillow, and in one of them he expired, at two o'clock P.M. on the sixth day of the disease.

*Dissection.*—On opening the cavity of the chest, the immediate cause of his death, as we had conjectured, was clearly ascertained to be inflammation of the heart; that viscus was considerably enlarged in size, and the surface every where covered with a layer of coagulable lymph, so loosely adhering as to yield to slight pressure with the finger. The liquor pericardii was more abundant than usual, and of a turbid reddish colour. The inflammation had not extended to the lungs; and all the other viscera, both in the abdomen and the thorax, were apparently healthy.

